# Physical inactivity as a policy problem: applying a concept from policy analysis to a public health issue

**DOI:** 10.1186/1478-4505-11-9

**Published:** 2013-03-07

**Authors:** Alfred Rütten, Karim Abu-Omar, Peter Gelius, Diana Schow

**Affiliations:** 1 Institute of Sport Science and Sport, University of Erlangen-Nürnberg, Gebbertstr. 123b, D-91058 Erlangen, Germany

**Keywords:** Context analysis, Health promotion, Physical activity, Policy problem, Policy process, Public health

## Abstract

Despite the recent rapid development of policies to counteract physical inactivity (PI), only a small number of systematic analyses on the evolution of these policies exists. In this article we analyze how PI, as a public health issue, “translates” into a policy-making issue. First, we discuss why PI has become an increasingly important public health issue during the last two decades. We then follow Guy Peters and conceptualize PI as a “policy problem” that has the potential to be linked to policy instruments and policy impact. Analysis indicates that PI is a policy problem that i) is chronic in nature; ii) involves a high degree of political complexity; iii) can be disaggregated into smaller scales; iv) is addressed through interventions that can be difficult to “sell” to the public when their benefits are not highly divisible; v) cannot be solved by government spending alone; vi) must be addressed through a broad scope of activities; and vii) involves interdependencies among both multiple sectors and levels of government.

We conclude that the new perspective on PI proposed in this article might be useful and important for i) describing and mapping policies to counteract PI in different contexts; ii) evaluating whether or not existing policy instruments are appropriate to the policy problem of PI, and iii) explaining the factors and processes that underlie policy development and implementation. More research is warranted in all these areas. In particular, we propose to focus on comparative analyses of how the problem of PI is defined and tackled in different contexts, and on the identification of truly effective policy instruments that are designed to “solve” the PI policy problem.

## Introduction

Public health scientists are increasingly adopting physical activity as a research topic. This trend is borne out in the literature. A Medline search reveals an almost fourfold increase over the past two decades in the number of articles containing the phrase “physical activity,” from 2,286 articles published between 1991 and 2000 to 8,863 articles published between 2001 and 2010.

Underlying the increased importance of physical activity as a concept are high levels of sedentary behaviors in many countries [1,2] and mounting evidence on the connection between physical inactivity (PI) and other public health issues like the obesity epidemic and increasing rates of non-communicable diseases, such as type 2 diabetes [3-5].

National and international public health policies that tackle PI are also rapidly developing. The WHO’s “Global Strategy on Diet, Physical Activity and Health” [6] sends a clear message about the importance of policies promoting physical activity. Additionally, numerous policy documents like the European Union’s Physical Activity Guidelines [7] and the Healthy People 2020 Objectives of the United States Department of Health and Human Services [8] convey strong support of physical activity promotion initiatives.

A systematic analysis of the evolution of physical activity policies would be highly warranted, particularly as evidence indicates that physical activity policies are either underdeveloped or require improvement in many countries [9-12]. To date, however, only a small number of systematic analyses address the evolution of physical activity policies. Most of the analyses that do exist are either collections of case studies [13], descriptive evaluations [11] or, as Breton and de Leeuw [14] note, public health investigations that lack inclusion of theoretical frameworks drawn from policy science disciplines.

Against this backdrop, we suggest that it is time to develop more specific and theory-based approaches to the analysis of policies counteracting PI. Such approaches should include exploration of the logic associated with the process of policy-making. Specifically, investigations should focus on how PI, as a public health issue, “translates” into an issue of policy-making, in other words how it is defined, viewed and solved as a policy problem. In his discussion of “The Problem of Policy Problems”, Peters proposes a set of seven variables that can be used to define and characterize policy problems [15].

This article first briefly indicates some key characteristics of PI as a public health issue. It then introduces the concept of policy problems and provides a preliminary description of PI in relation to policy problem characteristics. It concludes that viewing PI as a “policy problem” is an important contribution to research in several respects. First, it provides a useful framework that can help describe and map health promotion policies in different contexts. Second, it can assist in evaluating whether or not existing policy instruments used to promote physical activity are appropriate. Third, it may help investigate the factors and processes that underlie the development and implementation of policies counteracting PI.

### Physical inactivity as a public health issue

In the last two decades, PI has become increasingly recognized as an important public health issue. This section addresses five key characteristics that are integral to contextualizing PI as a public health issue. It then discusses how these characteristics affect perceptions and approaches to reducing PI and promoting physical activity. This latter dynamic has an influence on how PI is translated into a “policy problem” in various circumstances.

#### PI as key lifestyle component relating to the obesity epidemic and non-communicable diseases

PI’s increasing relevance as a public health issue is closely tied to its role relating to the obesity epidemic as well as non-communicable disease rates (NCDs) like type II diabetes and heart disease [16], which are on the rise [3,4]. In this context, physical activity is perceived as a key “healthy lifestyle” component that is associated with both individual agency (*e.g*. capabilities to be physically active) and structural issues (*e.g.* access to infrastructures) [17-19].

#### Stable and increasing prevalence of physical inactivity

Despite increasing efforts to promote physical activity, the prevalence of PI has remained rather stable in most developed nations [20,21]. It is even on the rise in developing nations because patterns of behavior similar to those in developed countries are being adopted (such as urbanization, car ownership) [22]. These dynamics have thrust the importance of engaging in physical activity onto the global stage and have resulted in newly formed partnerships and approaches to reduce PI [6].

#### Advent of the concept of HEPA

The advent of the HEPA concept (Health Enhancing Physical Activity [23]) in the mid-1990s directed researchers’ attention to “any form of physical activity that benefits health and functional capacity without undue harm or risk” [24]. The scope of fields in which PI might be reduced was expanded beyond sport and exercise to include a number of broader domains such as leisure-time, transportation, occupational work and household. These domains involve additional stakeholders and multiple sectors and levels of activities [25].

#### Multiple causes and ecological models of PI

PI is attributed to a number of determinants. Among them are age, educational attainment (demographic and biological factors), attitudes, enjoyment (psychological factors), social support from family and peers (social and cultural factors), and the availability of infrastructures and facilities (physical environment factors) [26]. Ecological models have been developed to cover the variety of, and interrelationships between, causal factors [27].

#### Multiple types and combinations of interventions to promote physical activity

There exists a variety of evidence-based interventions to tackle the public health issue of PI [28-31]. They include individual and group-based interventions (*e.g.* exercise classes), community-based interventions (*e.g*. interventions focused on broadly publicizing information over a longer period of time), mass media campaigns, health-care setting interventions (*e.g*. green prescriptions), policy interventions (*e.g*. daily physical education lessons required by school curricula) and environmental interventions (*e.g*. building sidewalks, sports facilities). Reviews of these interventions do indicate that a number of different strategies might need to be combined in order to effectively promote physical activity [32].

### Physical inactivity as a policy problem

There exists a substantial body of literature on the role of policy problems within the policy-making process. A major topic in this literature is agenda setting, in other words when and how do public issues become problems that are believed to require public action [33,34]. Another important discourse that has a strong social constructivist component pertains to policy problem definition, *i.e.* how different political actors perceive and frame problems according to their preferences and goals [35-37]. Several authors provide criteria for how to distinguish and characterize policy problem definitions [35,38]. However, this literature remains focused on the different perspectives various actors have on problems. By contrast, Peters [15] provides a point of view that is more concerned with the “nature” of policy problems, *i.e.* with attributes that are inherent in the problems themselves. Such a perspective is interesting because it directs attention to potentially successful problem solutions.

According to Peters, defining public issues such as PI as “policy problems” helps to “explicate the relationships between problems and instrument choice” [15], and may thus lead to a “conscious selection of instruments, as opposed to their selection merely on the basis of custom, familiarity and institutional inertia” [15]. Characterizing policy problems is a two-stage process. While the first stage involves “defining what the problem is about” [15], the second, and potentially more difficult stage involves “framing the problem for solution.” This stage also includes preparation of appropriate policy instruments.

Given the well-known relationship between implementing appropriate policy instruments and subsequent policy outcomes [15], further investigation of the second stage of policy problem definition seems to be crucial for both developing adequate theories about policies to counteract PI and for designing successful strategies for practical policy implementation. To date, however, such an investigation is neither a major topic of interest amongst policy analysts nor is it featured in the public health literature.

In the following discussion we engage in Peters’ second stage in Peters' second stage of policy characterization by applying his recommended set of seven variables to PI [15]. These variables clarify the i) “solubility”; ii) “complexity”; iii) “scale”; iv) “divisibility”; v) “monetarization”; vi) “scope of activity”; and vii) “interdependencies” of PI as a policy problem. By applying these variables, we can begin to identify some general features that distinguish PI from other policy problems. As discussed below, contextual factors may contribute to the way that PI is defined as a policy problem in different regions or nations. However, we argue that some basic features that characterize PI as a policy problem are applicable in all political contexts, as they flow from the nature of the social phenomenon of PI rather than from contextual particularities.

#### “Solubility”

The first variable introduced by Peters is “solubility”. Peters defines it as the degree to which policy problems can actually be solved. He suggests that acute problems may be solved within a “once and for all/finite” amount of time, while chronic problems are more likely to appear and reappear on policy agendas despite the fact that many attempts to provide solutions may have already been made (*e.g.* health and economic policy).

PI can be regarded as a chronic rather than an acute policy problem. In general, both national and supranational policies to counteract PI seem to have resulted in only modest increases in physical activity levels. For example, data gathered by the U.S. National Center for Health Statistics [21] indicate that sedentary lifestyles have remained virtually unchanged in the last 10 years. In addition, frequent calls for new policies in the area of PI underline the chronic nature of the policy problem [39]. As a result, much existing literature seems to support policy options that work to reduce PI rather than to eliminate it entirely.

According to Peters, chronic policy problems often result in two noteworthy consequences that affect successful policy implementation [15]. The first consequence pertains to sustainability, and refers to the need to develop policy procedures and monitoring that are able to deal with the policy problem on a long-term basis. Although national policies to counteract PI were developed in a number of countries in the last two decades, real-world time frames for policy implementation often remain rather short, and political efforts strongly vary over time. For example, in a historic comparison of physical activity policies in Australia and seven other countries, Bellew *et al.* found that only few countries were “committed to policy of more than three years duration” [10]. They concluded that “[n]either overseas nor in Australia was there evidence of success at national level in the clear delineation of coalition roles and responsibilities, matched with long term commitment, or evaluation of policy implementation” [10].

The second consequence pertains to the fact that such problems may require more than one type of solution. In order to address this, Peters suggests using policy instruments that are flexible and adaptable [15]. He suggests that instruments such as “command-and-control” regulations might be less appropriate for problems like PI than participatory and collaborative methods that allow for mutual adjustment involving a variety of stakeholders. For the field of PI, a joint expert meeting of WHO and the CDC highlighted the importance of stakeholder consultation for effective policy development [40], and research projects at both the community level [41] and the regional/national level [42] have provided empirical evidence for the usefulness of stakeholder involvement. In addition, built-in evaluation and quality management tools may contribute to the flexibility of policy instruments. Daugbjerg *et al.*’s analysis of 27 policy documents related to physical activity promotion in European countries is telling in this regard because it indicates that systematic evaluation is still a vision rather than a reality in relation to national policies counteracting PI [11].

#### “Complexity”

“Complexity” is the second variable Peters uses to characterize policy problems. Importantly, he makes a distinction between “political complexity” and two forms of “programmatic complexity” [15]. Political complexity refers to the “degree of difficulty in negotiating agreements among the parties involved” [15]. Programmatic complexity refers both to the “technical content” of a policy problem and to the number of “ways of conceptualizing the root causes of that problem” (multiple causation [15]).

PI can be characterized as a policy problem with a high degree of political complexity because it involves a variety of sectors, actors and interests. Its programmatic complexity appears comparably low at first sight, at least with respect to its “technical content” [15]. For example, the benefits of maintaining an active lifestyle (*e.g.* walking and biking during leisure time or for purposes of commuting to school or work) are easily understood by “the average citizen” [15]. PI becomes a comparatively complex issue, however, when considered in relation to different “models of multiple causation” [15]. These models are designed to address a large number of variables (*e.g*. individual, social and environmental) that may influence changes in sedentary behaviour. Various parties have been known to compete to win the adoption of their favoured models as “the most relevant” frameworks for use in the creation of physical activity policies and interventions. For instance, in her analysis of the Active Living policy in Canada, Bercovitz argued that the rhetoric of “active lifestyle” as a personal choice and individual responsibility was used to conceal structural health inequalities and the retreat of the welfare state from social responsibility for health in times of financial crisis [43].

Thus, political and programmatic complexities of a policy problem might also interact. Arguments about the superiority of different multiple causation models might result in political struggles about which interventions should be used to promote physical activity. For example, some political actors might call for mass media campaigns to address psychological factors associated with physical activity behaviour. Since the development of mass media campaigns might also reduce levels of inter-sectoral dependencies (they are more easily developed by a single policy sector), such campaigns might be seen as appropriate. Other political actors, however, might dispute the effectiveness of mass media campaigns and call instead for other kinds of programs. An important example is the CDC in the United States, which found evidence on the effectiveness of stand-alone mass media campaigns to promote physical activity to be insufficient [29] and recommended integrated community-based interventions with a focus on physical activity-friendly environments instead [28].

The consequences resulting from dealing with policies that maintain high levels of complexity coincide with consequences associated with other variables. For example, PI’s high level of political complexity may lead researchers to the same conclusions as those reached when analyzing PI’s “solubility”. To that end, researchers might conclude in both situations that participatory and collaborative approaches should be used in physical activity promotion policy processes because “policy design efforts should enable processes that are: (1) flexible enough to respond to varying interests; (2) understood by all those involved; (3) defined in terms of specific processes for overcoming stalemate and disagreement” [15]. High political complexity may also lead researchers to similar conclusions as those reached when analyzing causation. In this context, it may be adequate to apply multiple policy instruments to deal with the variety of variables that may be relevant for changing sedentary behaviour [15].

#### “Scale”

The question of scale refers to the degree to which a policy problem can be disaggregated into smaller components that are easier to deal with than the problem as a whole. PI does not represent a typical large-scale problem requiring an “all or nothing approach.” It is “susceptible to disaggregation into smaller scales” [15], and it may be dealt with by incrementally adjusting existing policy instruments. For example, the development of a physical activity-friendly environment does not require large spending at a single point in time but may be dealt with by various actors from different sectors and at different levels through multiple smaller budget allocations over an extended period of time. A point in case for susceptibility of PI to disaggregation is the Norwegian “Action Plan on Physical Activity” [44], which specified 108 separate measures in 13 action areas implemented over the course of five years under the auspices of eight different ministries. In conclusion, the scale of PI as a policy problem necessitates the use of certain policy designs already mentioned above, such as employing multiple and flexible policy instruments rather than focusing on “the one and only” solution.

#### “Divisibility”

The question of “divisibility” does not so much pertain to the disaggregation of problems into smaller sub-problems (see variable no. 3, “Scale”) but to the question of how costs and benefits relating to the solution of a policy problem are distributed [15]. For example, if a policy problem requires a collective solution *(i.e.* the costs are highly divisible) but only leads to benefits for a specified group (*i.e.* the benefits are almost indivisible), it is theorized to be more difficult to solve than problems that yield widely-distributed benefits.

With respect to PI, many interventions are targeted toward specific sub-populations. While there are often good reasons for this, policies promoting such interventions may be more difficult to “sell” to the public (especially when the target group in question, such as a socially disadvantaged population, does not have a lobby) than policies that support interventions directed at broader populations (*e.g.* health education programs and mass media campaigns). Another potential divisibility issue is that certain public initiatives to promote physical activity might yield benefits mostly for those who are already active (*e.g.* public subsidies for new sport facilities). A more “divisible” alternative might be the promotion of multi-use infrastructures or broader environmental interventions (*e.g.* walking and biking paths) that benefit a wider range of people.

The issue of divisibility of PI as a policy problem may also apply to potential collaboration and conflicts between different sectors, for example the sport and the health sector. Willingness of agencies to cooperate inter-sectorally may depend on the division of costs and benefits of potential actions between sectors. Bercovitz describes how the launch of the Active Living policy by the Canadian government in the early 1990s caused organizations from the fitness and amateur sport sector to become more involved in the health arena due to expected organizational benefits [43]. Conversely, Bellew *et al.* indicate that the Australian government’s reorientation of physical activity policy and funding towards elite sports in the early 2000s reduced inter-sectoral collaboration [10].

#### “Monetarization”

“Monetarization” refers to whether policy problems can be solved strictly by funding or if they require other forms of action, such as advocacy and education [15]. Many factors are known to impact physical activity behaviour, including value orientation. For example, cultural perceptions about the appropriateness of “sweating in public” or gender issues might inhibit physical activity behaviour in different nations [26]. Value orientations are unlikely to be changed by allocating financial resources alone. Instead, they may require application of a mix of educational and other strategies. This dynamic renders the policy problem of PI more difficult to deal with.

Experience from community-based interventions illustrates the limited monetarization of PI. For example, public funding was an important aspect in BIG project, which targeted women in difficult life situations in a German city [41]. Financial support enabled women to take part in a cooperative process to plan physical activity programs, and low fees were an important prerequisite for many women to participate in physical activity classes. At the same time, however, there were important barriers to physical activity that could not be removed by simply spending money. For example, the mixed-gender policy at municipal swimming pools prevented many migrant women from swimming. This problem was eventually solved by altering pool access policies and creating women-only pool hours.

#### “Scope of activity”

“Scope of activity” refers to the variety of activities and behaviours that “contribute to the creation of a problem” [15]. If the number of actors involved is comparably small and if the behaviours to be influenced are not too complex, direct regulatory governmental interventions designed to solve the problem might be more successful than if the situation involved a larger number of actors (individuals, organizations and sectors) and behaviours. As a consequence, the solution of policy problems with a broad scope of activity might call for policy instruments that are more sophisticated than ones like command-and-control regulations passed down by the government. In particular, multiple and flexible policy instruments (like the ones already indicated above) are likely to be appropriate in this context.

The scope of activity involved in counteracting PI is considerable. As such, it may require the use and development of interventions that attempt to change behaviour in large segments of the population as well as in organizations from public, volunteer and private sectors. Indeed, governments tend to initiate a number of different actions to deal with the broad scope of activities involved in promoting physical activity. National action plans commonly outline activities in a variety of areas, such as physical education in schools, campaigns to promote walking for leisure and transportation, and community partnerships. Examples include the Norwegian “Action Plan for Physical Activity” (13 action areas) [44], the “Blueprint for an active Australia” (10 key action areas) [45], the Brazilian “Strategic Action Plan to tackle NCDs” (5 Action Areas) [46] and the German “National Action Plan for Diet and Physical Activity (IN FORM)” (5 action areas) [47].

#### “Interdependencies”

Finally, “interdependencies” refer to the degree to which policy problems can be solved within one policy domain or by one lone government agency. Peters argues that the “degree of interdependence characterizing any particular problem influences the capacity of government to solve the problem, as well as the range of appropriate policy instruments” [15].

Regarding PI, one can observe a high degree of interdependencies that lead to the involvement of a variety of policy domains, agencies and levels of government: First, there is broad scientific evidence that substantiates the fact that the environment influences physical activity levels, and there is also evidence that substantiates the fact that environment-oriented interventions stimulate physical activity [48]. Thus, in efforts to develop more physical activity-friendly environments, actors in the public health domain may find themselves collaborating with policy actors who are responsible for parks and recreation, sport (political competence for sport facilities) and transportation/urban planning (political competence for biking and walking trails).

Second, in many countries, the main responsibility for developing and implementing policies related to PI rests at the regional and local levels. Both the United States and Canada serve as cases in point. In both nations, a considerable percentage of policy-making takes place at regional and local levels [49]. A complete analysis of PI-related policy processes in North America should consider the interaction and contribution of the various government levels.

Third, responsibility for policy development and implementation quite often extends beyond the public policy sector. For example, in Germany, sport clubs assume a central role in delivering health related physical activity programs.

The challenges of “interdependencies” already have been recognized in many policy documents. National physical activity action plans [44-46,50] emphasize the interdependency between different sectors and ministries. On the supranational and international level, the EU’s Physical Activity Guidelines [7] and WHO’s Global Strategy on Diet, Physical Activity and Health [6] call for the involvement of different policy sectors, for cooperation between the European, national, regional and local levels of government, and for including the public sector, non-governmental organizations and the private sector.

However, despite widespread recognition of the interdependency of PI during policy formulation, countries still often fail to apply adequate policy instruments for inter-sectoral and multilevel policy implementation. The Norwegian Action Plan, for instance, has been selected as a good practice example for physical activity policy in Europe on several occasions. However, a systematic evaluation of the plan revealed a lack of coordinated use of instruments towards common targets across sectors, inadequate adjustment to regional and local actors (*e.g.* NGOs), and insufficient assignment of long-term responsibilities and resources [51].

## Conclusions

This article proposed a change in approach to physical activity policy research by viewing PI not only as public health issue but also as a policy issue. It applied the two-stage process developed by Peters to characterize PI as a policy problem [15]. It demonstrated that PI is a policy problem that: i) is chronic in nature; ii) involves a high degree of political complexity; iii) can be disaggregated into smaller scales; iv) is addressed through interventions that can be difficult to “sell” to the public when their benefits are not highly divisible; v) cannot be solved by government spending alone; vi) must be addressed through a broad scope of activities; and vii) involves interdependencies among multiple sectors and levels of government.

Conceptualizing PI as a “policy problem” is more than just a theoretical exercise. We argue that it can provide an important contribution to research in several respects:

First, it provides a framework that can help describe and map policies to counteract PI in different contexts. It is sensitive to the specificities of both PI as a public health issue and generic characteristics of policy problems. Instead of mapping PI-related policies along a set of general dimensions such as nation, sector, and target group, defining PI as a policy problem directs analysis towards truly relevant categories (*i.e.* Peters’ characteristics) of policymaking and thus allows for more development of refreshing perspectives regarding need and choice of policy instruments.

Second, the framework may be employed to evaluate whether or not existing policy instruments to promote physical activity are really appropriate given the characterization of PI as a policy problem. Policy instruments that fail to address the “nature” of the policy problem are less likely to be effective than instruments that do.

As outlined above, potentially effective policy instruments might be complex, flexible and sustainable, have built-in evaluation and quality management tools, work incrementally rather than attempting to provide a “once and for all” solution, take into account the complex interdependencies between sectors and work at the appropriate levels of government. By contrast, command-and-control regulations or policy instruments that focus on spending alone might be less suited to solve the policy problem of PI. The policy problem concept could thus be an important tool for advising policy-makers on selecting and implementing more appropriate instruments to promote physical activity in the future.

From a scientific point of view, the proposed concept could also be used to go one step further, in other words to explain why policies counteracting PI were developed in country A but not in country B, why they were successful in one case but not in another, and why certain policy instruments were chosen. To do so, one would have to investigate the processes underlying policy-development as well as the contextual factors that shape the political landscape in a certain nation, region or community.

We believe that further research in two areas might be particularly fruitful: comparative research and research on policy instruments.

Defining PI as a policy problem using the variables suggested by Peters is an important first step to highlighting the general characteristics of PI as opposed to the characteristics of other major policy problems. Against this conceptual background, it is worthwhile to further investigate how and why the specific definition of PI as a policy problem varies in different policy environments. One important factor of influence pertains to the processes employed in the making of policies (*e.g*. agenda setting procedures). Several theoretical approaches might be useful in analyzing these processes, for example: i) the Multiple Streams framework [52] which theorizes how policy entrepreneurs may use windows of opportunity to put their issues on the political agenda; ii) the Institutional Rational Choice framework [53] which aims to explain the influence of institutional rules and resources on the behaviour of political actors; iii) the Advocacy Coalition framework [54] which focuses on how coalitions of political actors form around certain policy problems; and iv) the Analysis of Determinants of Policy Impact model [55] which links policy problems to policy determinants and policy outcomes.

As shown above, we argue that there are a number of aspects that characterize the policy problem of PI in general, *i.e.* regardless of regional or national contexts. Nonetheless, the specific “expression” of some of the variables that define the problem may be shaped by contextual factors. For example, “interdependencies” has different expressions in Germany, which has separate ministries for health and sport, and in the Netherlands, where sport and physical activity are part of the portfolio of the Ministry of Health. This, among other things, has led to the development of dedicated organizational structures to deal with the policy problem of PI, such as the Netherlands Institute for Sport and Physical Activity, which pursues and integrated agenda to promote sport, physical activity and health.

The structure of political systems may also be of particular relevance. Distinguishing between presidential and parliamentary systems, between centralized and federalist states, or between different health care [56] and welfare systems [57] might be helpful in explaining differing definitions of PI as a policy problem. For instance, it may be worth investigating whether liberal welfare state models in North America lead to definitions of PI as a policy problem that are more focused on individual behaviour than in the corporatist and social democratic welfare regimes of Central and Northern Europe [57]. Other relevant context variables include economic, social, and cultural factors [58,59] as well as demographic, geographic, climatic and natural characteristics of a given community, region or country.

While these contextual factors may influence the particular expression of the nature of a policy problem under specific circumstances, one must also bear in mind that the way a policy problem is defined in a political arena may also depend on strategic decisions by the actors involved in the policy-making process. The literature on the politics of problem definition mentioned above will be useful to analyze this particular aspect of policy problems. In extreme cases, policy problem definitions may even contradict the nature of the policy problem, such as when political actors define PI as a policy problem that can be handled by a single sector because they want to prevent the involvement of other sectors with competing interests.

Future research might build on the above-mentioned considerations to further specify the nature and definition of PI as a policy problem in certain contexts (with a particular focus on, but not limited to, different countries) and to compare select contexts with respect to their similarities and differences, thus contributing to a deeper understanding and better explanations about how exactly policy processes and contexts shape the definition of policy problems.

Besides comparative research on the general and context-specific definitions of PI as a policy problem, an important next step would be to investigate which solutions, or policy instruments, might be best suited to solve the problem. The ultimate goal of such research would then be to make physical activity promotion policy “truly” evidence-based, as opposed to simply applying the logic of evidence-based interventions to the fundamentally different realm of policy-making [60].

Of course, as Wildavsky and other “realist policy analysts” have emphasized, policy-making may not follow “the rational paradigm”, where problems are defined first and the most appropriate solutions are selected afterwards. In real-world policy-making, “solutions often search for problems” [61]. For example, mass media information campaigns, although less effective for promoting behavioural changes, are popular physical activity promotion interventions in many countries. Government agencies usually have great experience and expertise in “public relations”, and political actors are very much aware of the importance of mass media. Thus, an information campaign (maybe even featuring a well-known role model) may be considered a promising, proven and tested “solution” in political terms. In order to apply such a solution to PI as a policy problem, certain characteristics indicated above may be highlighted (for example the role of psychological factors of physical activity behaviour as one part of its programmatic complexity) while others may not (such as environmental factors as another part of its programmatic complexity). For this reason, it is also important for researchers to choose appropriate tools of making their knowledge on PI as a policy problem available to policy-makers and thus assist the development of policies to solve the problem. Most scholars agree that traditional linear models of “knowledge transfer” are inappropriate [62], and that research is “most likely to influence policy development through an extended process of communication and interaction” [63]. Suitable approaches suggested include “knowledge brokering” [64,65], “nexus theories” [66] or “interactive knowledge-to-action” approaches [42].

This last point also directs attention to an interesting final theoretical consideration, namely the link between policy instruments and physical activity promotion interventions. It can be assumed that certain interventions may only be successfully implemented if they are supported by corresponding policies. For example, an infrastructural intervention involving multiple sectors can only be successful if governmental agencies from all of the sectors are involved in shaping physical activity promotion policy. If this is not the case, inter-sectoral interventions might fail even if they have been shown to be effective in other contexts. Thus, a thorough understanding of PI as a policy problem might not only be useful in identifying better policy instruments, but it also may help achieve a better match between interventions and policies in future physical activity promotion efforts.

## Abbreviations

PI: Physical inactivity.

## Competing interests

The authors declare that they have no competing interests.

## Authors’ contributions

AR: Designed the study, developed the outline, contributed to the analysis, and contributed to the writing and revision of the manuscript. KAO: Revised the outline, contributed to the analysis, and contributed to the writing and revision of the manuscript. PG: Revised the outline, contributed to the analysis, and contributed to the writing and revision of the manuscript. DS: Revised the outline, contributed to the analysis, and contributed to the writing and revision of the manuscript. All authors read and approved the final manuscript.
